# TRPV1 protects renal ischemia-reperfusion injury in diet-induced obese mice by enhancing CGRP release and increasing renal blood flow

**DOI:** 10.7717/peerj.6505

**Published:** 2019-02-27

**Authors:** Beihua Zhong, Shuangtao Ma, Donna H. Wang

**Affiliations:** 1 Division of Nanomedicine and Molecular Intervention, Department of Medicine, Michigan State University, East Lansing, MI, USA; 2 Neuroscience Program, Michigan State University, East Lansing, MI, USA; 3 Cell & Molecular Biology Program, Michigan State University, East Lansing, MI, USA

**Keywords:** TRPV1, Renal ischemia-reperfusion, Renal blood flow, Western diet, Calcitonin gene-related peptide

## Abstract

**Background:**

Obesity is a major risk factor for end-stage renal disease. Using transient receptor potential vanilloid 1 knockout (TRPV1^−/−^) mice, we tested the hypothesis that TRPV1 protects against obesity-induced exacerbation of renal ischemia-reperfusion (I/R) injury.

**Methods:**

TRPV1^−/−^ and wild-type (WT) mice were fed a chow or Western diet (WD) for 22–23 weeks. After that, mice were subjected to renal I/R injury, and renal cortical blood flow (CBF) and medullary blood flow (MBF) were measured.

**Results:**

The Western diet significantly increased body weight and fasting blood glucose levels in both TRPV1^−/−^ and WT mice. WD-induced impairment of glucose tolerance was worsened in TRPV1^−/−^ mice compared with WT mice. WD intake prolonged the time required to reach peak reperfusion in the cortex and medulla (both *P* < 0.05), decreased the recovery rate of CBF (*P* < 0.05) and MBF (*P* < 0.05), and increased blood urea nitrogen, plasma creatinine, and urinary 8-isoprostane levels after I/R in both mouse strains, with greater effects in TRPV1^−/−^ mice (all *P* < 0.05). Renal I/R increased calcitonin gene-related peptide (CGRP) release in WT but not in TRPV1^−/−^ mice, and WD attenuated CGRP release in WT mice. Moreover, blockade of CGRP receptors impaired renal regional blood flow and renal function in renal I/R injured WT mice.

**Conclusion:**

These results indicate that TRPV1 plays a protective role in WD-induced exacerbation of renal I/R injury probably through enhancing CGRP release and increasing renal blood flow.

## Introduction

Obesity is a major risk factor for diabetes and hypertension (Hall, 2003; Steinberger & Daniels, 2003), which together account for 70% of all cases of end-stage renal disease (Collins et al., 2005; Griffin, Kramer & Bidani, 2008). Ischemia-reperfusion (I/R) injury is a common cause of acute kidney injury (AKI) in a variety of clinical settings (Lameire, Van Biesen & Vanholder, 2006). AKI occurs in approximately 7% of hospitalized patients (Nash, Hafeez & Hou, 2002) and is associated with an overall mortality of 40–60% in critically ill patients (Uchino et al., 2005). Western diet (WD) intake leads to obesity, aggravates chronic inflammation, and enhances acute renal ischemic injury (Kelly, Burford & Dominguez, 2009).

Transient receptor potential vanilloid 1 (TRPV1) is a ligand-gated non-selective ion channel, which can be activated by multiple stimuli including noxious heat, low pH, and chemical irritants such as capsaicin (Caterina et al., 2000; Julius & Basbaum, 2001). TRPV1 is primarily expressed in unmyelinated C-fibers and thinly myelinated Aδ-afferent nerve fibers, which innervate internal organs including the kidneys (Caterina et al., 2000; Julius & Basbaum, 2001). TRPV1 activation causes release of neuropeptides, including calcitonin gene-related peptide (CGRP) and substance P with potent vasodilatory effects (Wang, 2005) as well as natriuretic and diuretic effects (Arendshorst, Cook & Mills, 1976; Shekhar et al., 1991). The kidney is innervated by a dense network of CGRP-positive sensory nerves (Chai et al., 1998). Unlike many other vasodilators, CGRP increases renal blood flow and glomerular filtration (Shekhar et al., 1991). TRPV1 deficiency results in enhanced I/R injury (Huang et al., 2009), suggesting that TRPV1 plays an important role in renal injury (Levite et al., 1998, Okajima & Harada, 2006).

Transient receptor potential vanilloid 1 is closely linked to and interacts with obesity (Gram, Holst & Szallasi, 2017). Obesity impairs sensory nerves-mediated vasodilatation (Davidson et al., 2010, Haddock & Hill, 2011). Baskaran et al. (2017) reported that diet-induced obesity suppressed TRPV1 expression in adipose tissue. In addition, Kentish et al. (2015) demonstrated that ganglionic TRPV1 was inactivated in obese mice induced by high-fat diet. Moreover, TRPV1 deficiency exacerbates diet-induced obesity and insulin resistance (Lee et al., 2015). Thus, WD intake could impair TRPV1 expression or function, leading to a more severe renal I/R injury, and TRPV1 deficiency could exacerbate renal I/R injury. In this study, we tested the hypothesis that WD intake-induced obesity exacerbates renal I/R recovery and that TRPV1 ablation enhances renal perfusion impairment and subsequently deteriorates the recovery of renal function.

## Materials and Methods

### Feeding studies and drugs

The male TRPV1 gene knockout (TRPV1^−/−^) strain B6.129S4-TRPV1^tm1Jul^ and wild-type (WT) strain C57BL/6J mice (Jackson Laboratory, Bar Harbor, Maine) were used. Body weight was monitored from 3 to 23 weeks of age while both strains of mice had free access to chow diet (con; 8664; Harlan Teklad, Madison, WI, USA) or (WD; 42% kcal from fat; 88,137, Harlan Teklad) and tap water during the experiments. Mice were maintained in a normal light/dark cycle. Food intake and body weight were measured once a week. All experimental procedures were approved by the Institutional Animal Care and Use Committee of Michigan State University (08/14-141-00).

### Intraperitoneal glucose tolerance testing

Experimental mice were fasted (deprived of food but giving water) for 15 h, after which glucose (two g/kg body weight) was administered via intraperitoneal injection to conscious mice. Tail vein blood was sampled for glucose determination using an Accu-Chek glucose meter (Roche Diagnostics, Mannheim, Germany) at 0, 30, 60 and 120 min after glucose administration. The areas under the curve (AUC) for glucose tolerance were calculated according to the trapezoidal rule from glucose measurements at 0, 30, 60, and 120 min. Glucose tolerance was defined as AUC *vs* time curve calculated with the trapezoidal rule.

### Western blot analysis

Proteins were extracted from kidney tissues with lysis buffer containing protease inhibitors (Sigma, St. Louis, MA, USA). Total proteins were separated on a 10% sodium dodecyl sulfate -polyacrylamide gel, and transferred to a polyvinylidene difluoride membrane. The membranes were blocked 1 h at room temperature in 5% non-fat milk washing solution (50 mM Tris-HCl, 100 mM NaCl, 0.1% Tween 20, pH 7.5). Then the membranes were incubated with rabbit anti-TRPV1 polyclonal IgG (1:1,000, ab6166; Abcam, Cambridge, MA, USA) and anti-rabbit IgG-HRP (1:5,000, Santa Cruz Biotechnology, Dallas, TX, USA). The membranes were visualized with an enhanced chemiluminescence detection system (ECL; Amersham Biosciences, Piscataway, NJ, USA) and exposed to films (Hyperfilm-ECL; Amersham Pharmacia Biotech, Little Chalfont, UK). The densities were determined using Image J 1.46 (NIH), and normalized to the total amount of glyceraldehyde 3-phosphate dehydrogenase loaded in each well.

### Urinary 8-isoprostane analysis

At the end of 23-week-diet treatment, mice were placed in mouse metabolic cages for 24 h urine collection. Urinary 8-isoprostane levels were determined using a kit (Cayman Chemical, Ann Arbor, MI, USA).

### Renal ischemia-reperfusion model

Experimental mice were anesthetized with ketamine (80 mg/kg, i.p.) and xylazine (10 mg/kg, i.p.) and were placed on a heating pad to maintain body temperature at 37 °C. Mice received an intravenous infusion of a 0.9% sodium chloride solution containing 1% bovine serum albumin at a rate of two ml/100g/h throughout the experiment (Pechman et al., 2009). After surgical preparation, the kidney was placed in a clay cup without exerting tension on renal vessels. Cortical (CBF) and medullary (MBF) blood flows were measured simultaneously using a dual-channel Laser-Doppler flowmeter (Periflux 5000, Perimed, Jarfalla, Sweden). The probe was placed perpendicular to the surface of the cortex to measure CBF and MBF was measured by a probe inserted into the outer medulla at a depth of three to four mm. The position of the probe in the outer medulla was verified at the end of each experiment by dissecting the kidney. For certain groups, CGRP_8–37_ (20 nmol/kg/min, i.v.) was given during the renal I/R period. Baseline CBF and MBF were obtained after 30–60 min equilibration. Both renal vessels were then clipped with a microaneurysm clamp for 40 min and then released for 3 h. The CBF and MBF were obtained and analyzed by Perisoft (Perimed, Järfälla, Sweden). The following parameters were analyzed: recovery rate (percent change of reperfusion values from baseline); and time to peak reperfusion, that is, time elapsing from release of clamp to peak reperfusion of CBF and MBF. At the end, the mice were euthanized; urine, blood and kidney samples were obtained (Roman, Mattson & Cowley, 2001, Shi et al., 2007).

### Measurement of urinary lactate dehydrogenase

After reperfusion, urine was collected through a catheter placed in the urinary bladder, and samples were kept at −80 °C until analysis. Lactate dehydrogenase (LDH) was measured using a LDH Activity Assay Kit (Biovision, San Francisco, CA, USA).

### Assessment of renal function

Renal function was assessed by measuring plasma creatinine and urea concentration. Plasma samples were collected and measured by using standard assay kits (**Biovision**).

### Measurement of CGRP

Plasma was collected, and then CGRP were purified and analyzed using a rat CGRP radioimmunoassay kit. The assay was performed as recommended by the supplier (Peninsula Laboratories Inc., Belmont, CA, USA).

### Statistical analysis

All values are expressed as mean ± SEM. Differences among groups were performed by the one-way analysis of variance followed by the Tukey-Kramer multiple comparison test. Differences between two groups were performed by *t*-tests. The results were considered statistically significant at *P* < 0.05.

## Results

### Knockout of TRPV1 exacerbated WD-induced glucose intolerance and oxidative stress

**Figure 1:**
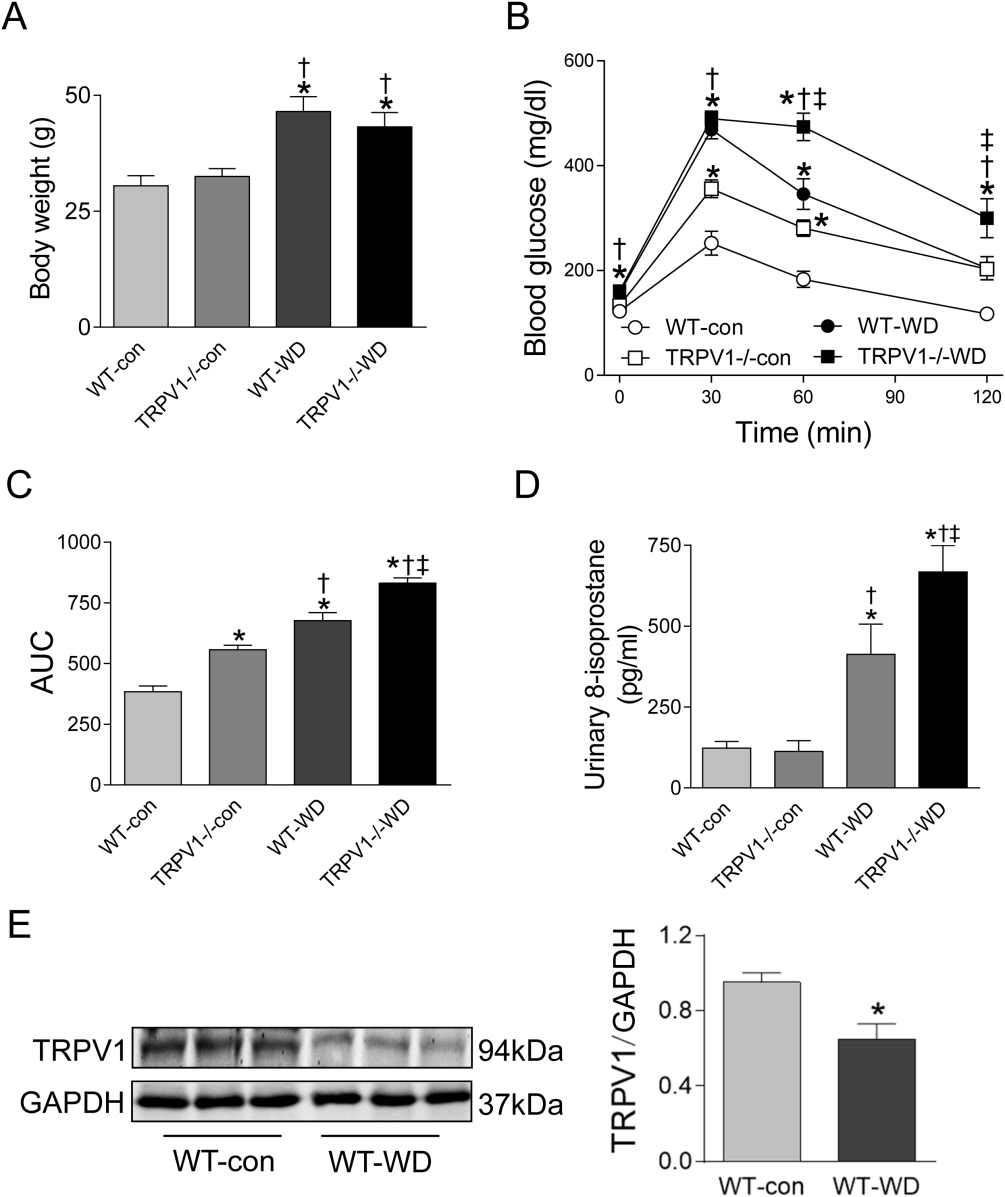
Body weight, glucose tolerance, oxidative stress, and TRPV1 expression. (A) Body weight was measured in WT and TRPV1^−/−^ mice after feeding chow (con) or Western diet (WD) for 23 weeks. (B) At 23 weeks, fasting plasma glucose was measured, and intraperitoneal glucose tolerance test was performed. (C) Mean area under the curves (AUC) of glucose was calculated. (D) Urinary 8-isoprostane levels were analyzed. Values are mean ± SEM, *n* = 6, *P < 0.05 *vs* WT-con, ^†^*P* < 0.05 *vs* TRPV1^−/−^-con, ^‡^*P* < 0.05 *vs* WT-WD. (E) Protein expression of TRPV1 in the kidney of WT mice fed with con or WD was measured by Western blotting and normalized by GAPDH. Values are mean ± SEM, *n* = 5, *P < 0.05 *vs* WT-con.

At 23 weeks, WD intake increased body weight in both WT and TRPV1^−/−^ mice when compared with chow diet groups (both *P* < 0.01), however, there were no differences between WT and TRPV1^−/−^ mice fed with either chow or WD (Fig. 1A). Similarly, fasting plasma glucose levels were significantly higher in WD groups in both strains (*P* < 0.01), and there was no different between WT and TRPV1^−/−^ mice (Fig. 1B). In contrast, WD-induced impairment of glucose tolerance was exacerbated in TRPV1^−/−^ mice compared with WT mice (*P* < 0.01, Figs. 1B and 1C). WD intake increased urinary 8-isoprostane levels in both strains (both *P* < 0.01), with a further increase in TRPV1^−/−^ mice compared with WT mice (*P* < 0.01, Fig. 1D). Interestingly, WD significantly decreased the expression of TRPV1 in the kidney of WT mice (*P* < 0.05, Fig. 1E).

### Knockout of TRPV1 exacerbated WD-induced impairment of renal blood flow recovery

**Figure 2:**
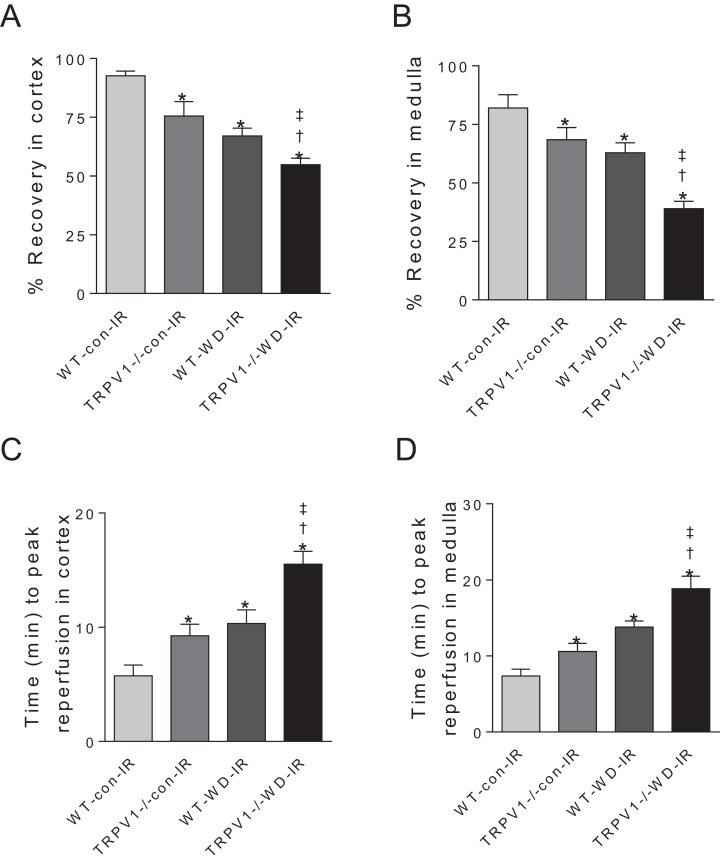
Recovery rates of renal blood flow and time to peak reperfusion. After 23-week-diet intervention, renal blood flow in cortex and medulla was measured before and after I/R injury, recovery rates (percent change of steady-state reperfusion values from baseline values) (A and B) and time to peak reperfusion (C and D) were calculated in WT and TRPV1^−/−^ mice on chow (con) or Western diet (WD). Values are mean ± SEM, *n* = 6–7, *P < 0.05 *vs* WT-con-IR, ^†^*P* < 0.05 *vs* TRPV1^−/−^-con-IR, ^‡^*P* < 0.05 *vs* WT-WD-IR.

After I/R, TRPV1^−/−^ mice on chow diet had significantly decreased recovery rates of CBF and MBF and increased time to peak reperfusion compared with WT mice on chow diet, suggesting that TRPV1 ablation impairs renal I/R recovery (Fig. 2). As expected, WD intake decreased recovery rates of CBF and MBF and increased time to peak reperfusion after renal I/R injury in both strains, with greater decreases in recovery rates and a further increase in the time to peak reperfusion in TRPV1^−/−^ mice (Fig. 2).

### Knockout of TRPV1 exacerbated WD-induced renal dysfunction

**Figure 3:**
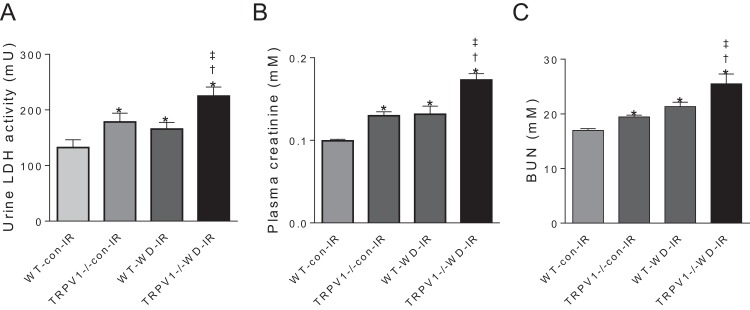
Renal injury and function. After 23-week-diet intervention and renal I/R injury, urinary lactate dehydrogenase (LDH) activity (A), plasma creatinine (B), and blood urea nitrogen (BUN), (C) were measured in WT and TRPV1^−/−^ mice. Values are mean ± SEM, *n* = 6–7, *P < 0.05 *vs* WT-con-IR, ^†^*P* < 0.05 *vs* TRPV1^−/−^-con-IR, ^‡^*P* < 0.05 *vs* WT-WD-IR.

In mice subjected to renal I/R injury, WD intake increased LDH activity, plasma creatinine and blood urea nitrogen (BUN) in both strains (all *P* < 0.01), with further increases in TRPV1^−/−^ mice compared with WT mice (all *P* < 0.01, Fig. 3).

### CGRP contributes to renal I/R recovery

**Figure 4:**
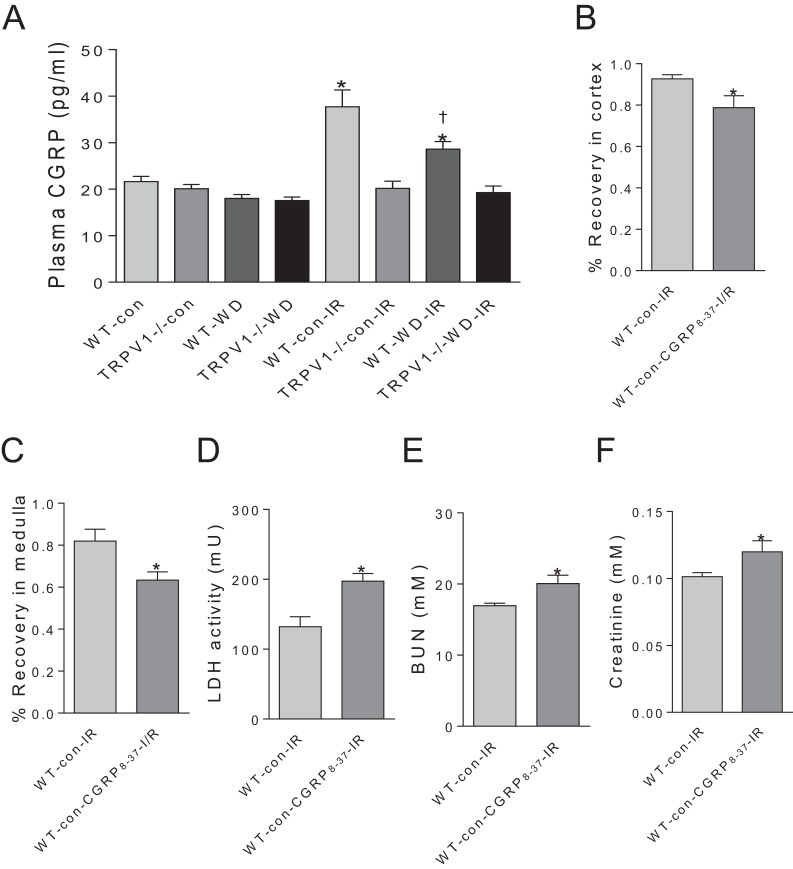
Effects of CGRP _8–37_ on renal blood flow and function. (A) Plasma CGRP was measured in WT and TRPV1^–/–^ mice on chow (con) or Western diet (WD) for 23 weeks, before and after renal I/R injury. Values are mean ± SEM, *n* = 6–7, *P < 0.05 *vs* WT-con, †*P* < 0.05 *vs* WT-con-IR. Recovery rates of CBF (B) and MBF (C), urinary LDH (D), BUN (E), and plasma creatinine (F) in WT mice on chow diet (con) with or without treatment of CGRP_8–37_. Values are mean ± SEM, *n* = 6–7, *P < 0.05 *vs* WT-con-IR.

Plasma CGRP levels have no significant differences between WT and TRPV1^−/−^ mice with either chow or WD diet. Renal I/R increased CGRP release in WT mice but not in TRPV1^−/−^ mice. Moreover, WD attenuated I/R-induced CGRP release in WT mice (Fig. 4A). In WT mice on chow diet, CGRP_8–37_ leaded to significant decreases in recovery rates of CBF and MBF and a remarkable increase in the time to peak reperfusion. Moreover, CGRP_8–37_ treatment increased urinary LDH, BUN and plasma creatinine levels in WT mice (Figs. 4B–4F).

## Discussion

In the present study, we found that WD intake caused glucose intolerance, oxidative stress, and impaired recovery of renal blood flow and renal function after I/R injury, which were exacerbated in TRPV1^−/−^ mice compared to WT mice. Renal I/R increased CGRP release in WT mice, which was blunted by WD intake. Blockade of the CGRP receptors impaired renal regional blood flow and renal function after I/R in WT mice. In contrast, CGRP release was not changed in TRPV1^−/−^ mice after renal I/R injury.

Transient receptor potential vanilloid 1 plays an important role in diet-induced obesity and glucose intolerance (Lee et al., 2015). We found that TRPV1^−/−^ mice had glucose intolerance even on a control diet, while urinary 8-isoprostane was normal compared to WT mice. Previous studies demonstrated that TRPV1 activation might induce insulin secretion (Mori et al., 2018). Therefore, TRPV1 deficiency-induced glucose intolerance is likely attributed to impaired insulin secretion rather than oxidative stress. This may be also a reason why diet-induced glucose intolerance was exacerbated in TRPV1^−/−^ mice.

Overall decrease in renal blood flow contributes to diminished glomerular filtration rate (GFR) in ischemic AKI (Alejandro et al., 1995). Our previous studies showed that activation of TRPV1 increased GFR and renal excretory function (Li & Wang, 2008), suggesting that TRPV1 ablation could further decreases GFR and renal excretory function during I/R. Other studies showed that TRPV1 activation enhanced endothelium-dependent vascular relaxation (Yang et al., 2010) and WD intake impaired endothelial function (Vogel, Corretti & Plotnick, 1997), suggesting that TRPV1 ablation could decrease renal blood flow recovery after renal I/R through directly impairing endothelium-dependent relaxation.

Transient receptor potential vanilloid 1 expression and function could be suppressed in obesity (Kentish et al., 2015, Baskaran et al., 2017). We found that renal TRPV1 expression was decreased in obese mice. TRPV1 deficiency worsened renal ischemic injury, suggesting that TRPV1 plays a protective role in renal I/R injury. TRPV1 mediates CGRP release during renal I/R. The decreased CGRP release in WD-fed WT mice compared to control diet-fed WT mice may contribute to the worsened renal I/R injury. CGRP-positive sensory nerves are widely expressed in the kidney (Chai et al., 1998). CGRP is a powerful vasodilatory neuropeptide (Kawasaki et al., 1988). Renal I/R increased CGRP release in WT mice, and CGRP_8–37_ impaired renal blood flow and renal function after I/R in WT mice, suggesting a protective role of CGRP in renal I/R recovery. It has been reported that renal ischemia enhanced renal sympathetic nerve activity (SNA), leading to a more severe ischemic AKI (Fujii et al., 2003). Therefore, CGRP may play a protective role in renal I/R via its vasodilatory effect or inhibition of renal SNA (Ralevic, Karoon & Burnstock, 1995, Oh-hashi et al., 2001). In addition to its well-recognized cardiovascular effects, CGRP also plays an important role in neuro-immune interactions (Reichlin, 1993). CGRP inhibits the ability of macrophages to produce H_2_O_2_ (Nong et al., 1989). CGRP-null mice have worsened inflammatory responses (Bowers et al., 2005). Thus, CGRP may protect against renal I/R injury via inhibiting inflammatory responses. Taken together, these results indicate that WD intake and TRPV1 ablation impair renal regional blood flow and renal function, at least in part, through decreasing release of CGRP.

Ischemia-reperfusion may increase endogenous TRPV1 agonists, such as 20-HETE analogues, which have been shown to increase renal blood flow in a dose-dependent manner (Oyekan, 2005). Furthermore, 20-HETE analogues have protective effects in experimental renal I/R (Regner et al., 2009). TRPV1 ablation or WD intake might impaired beneficial effects of endogenous TRPV1 agonists during I/R, leading to decreased renal blood flow and renal dysfunction.

## Conclusion

In summary, these results suggest that TRPV1 could play a protective role in renal I/R injury in obesity possibly via enhancing renal blood flow through increasing CGRP release.

## Supplemental Information

10.7717/peerj.6505/supp-1 Supplemental Information 1 Raw data that were used for data analyses and preparation for Figure 1, Figure 2, Figure 3, and Figure 4.

## Additional Information and Declarations

### Data Availability

The following information was supplied regarding data availability: The raw data are available in Data S1.
